# Structural and biophysical studies of new l-asparaginase variants: lessons from random mutagenesis of the prototypic *Escherichia coli* Ntn-amidohydrolase

**DOI:** 10.1107/S2059798322005691

**Published:** 2022-06-28

**Authors:** Joanna I. Loch, Agnieszka Klonecka, Kinga Kądziołka, Piotr Bonarek, Jakub Barciszewski, Barbara Imiolczyk, Krzysztof Brzezinski, Mirosław Gilski, Mariusz Jaskolski

**Affiliations:** a Department of Crystal Chemistry and Crystal Physics, Faculty of Chemistry, Jagiellonian University, Krakow, Poland; b Department of Physical Biochemistry, Faculty of Biochemistry, Biophysics and Biotechnology, Jagiellonian University, Krakow, Poland; c Institute of Bioorganic Chemistry, Polish Academy of Sciences, Poznan, Poland; d Department of Crystallography, Faculty of Chemistry, A. Mickiewicz University, Poznan, Poland

**Keywords:** l-asparaginases, Ntn-hydrolases, random mutagenesis, leukemia

## Abstract

This work reports the results of random mutagenesis of the *Escherichia coli* class 2 l-asparaginase EcAIII belonging to the Ntn-hydrolase family. New variants of EcAIII were studied using structural, biophysical and bioinformatic methods. Activity tests revealed that the l-asparaginase activity is abolished in all analyzed mutants with the absence of Arg207, but some of them retained the ability to undergo the autoproteolytic maturation process. The results of spectroscopic studies and the determined crystal structures showed that the EcAIII fold is flexible enough to accept different types of mutations; however, these mutations may have a diverse impact on the thermal stability of the protein. The conclusions from the experiments are grouped into six lessons focused on (i) the adaptation of the EcAIII fold to new substitutions, (ii) the role of Arg207 in EcAIII activity, (iii) a network of residues necessary for autoprocessing, (iv) the complexity of the autoprocessing reaction, (v) the conformational changes observed in enzymatically inactive variants and (vi) the cooperativity of the EcAIII dimer subunits. Additionally, the structural requirements (pre-maturation checkpoints) that are necessary for the initiation of the autocleavage of Ntn-hydrolases have been classified. The findings reported in this work provide useful hints that should be considered before planning enzyme-engineering experiments aimed at the design of proteins for therapeutic applications. This is especially important for l-asparaginases that can be utilized in leukemia therapy, as alternative therapeutics are urgently needed to circumvent the severe side effects associated with the currently used enzymes.

## Abstract 2

Random mutagenesis at designated sites of the l-asparaginase EcAIII was combined with crystal structure analysis and *AlphaFold*2 predictions to dissect the enzyme-maturation and catalytic processes.

## 1. Introduction

l-Asparaginases hydrolyze l-asparagine to l-aspartic acid and ammonia. Based on their amino-acid sequences and the architecture of the protein fold, l-asparaginases are divided into three classes (Loch & Jaskolski, 2021 ▸; da Silva *et al.*, 2022 ▸): class 1 (bacterial-type), class 2 (plant-type) and class 3 (*Rhizobium etli*-type) (Loch *et al.*, 2021 ▸). The prototypic members of class 1 include the *Escherichia coli* enzyme EcAII and its homolog from *Erwinia chrysanthemi* 1 (ErAII), which are used to treat acute lymphoblastic leukemia (ALL; Sugimoto *et al.*, 2015 ▸). EcAII and ErAII have high substrate affinity (*K*_m_ in the micromolar range), which makes them efficient scavengers of l-asparagine in the bloodstream, and thus very potent antileukemic drugs (Kotzia & Labrou, 2009 ▸; Maggi *et al.*, 2017 ▸). However, their administration is not free from severe side effects. A number of experiments have been undertaken to improve the properties of therapeutic l-asparaginases (Nguyen *et al.*, 2016 ▸; Lopes *et al.*, 2017 ▸; Aghaeepoor *et al.*, 2018 ▸; Maggi *et al.*, 2017 ▸; Verma *et al.*, 2014 ▸; Lu *et al.*, 2019 ▸; Kotzia & Labrou, 2009 ▸; Rigouin *et al.*, 2017 ▸). However, none of the improved variants could be introduced into clinical practice. Therefore, it seems reasonable to search for novel biotherapeutics among l-asparaginases from other classes.

**Figure 1:**
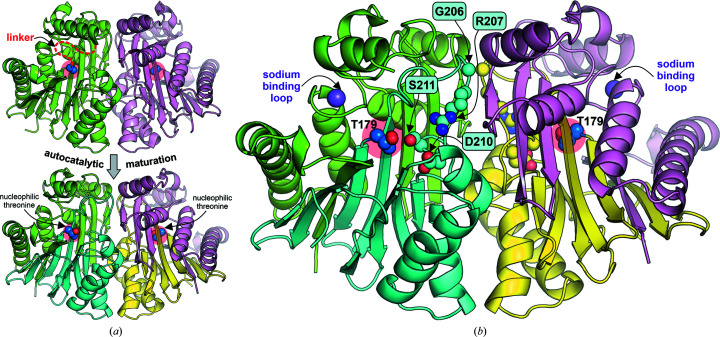
Autocatalytic maturation and key residues of EcAIII. (*a*) In the autocatalytic maturation the inactive precursor of EcAIII (PDB entry 2zak; chain *A*, green; chain *B*, pink) is cleaved into α/β subunits (PDB entry 2zal; α subunits, green/pink; β subunits, cyan/yellow). PDB entry 2zak carries the T179A mutation. In this process, the nucleophilic Thr179 (marked by a red circle) is liberated, while the linker (red dashed line) is partly degraded. (*b*) Residues subjected to random mutagenesis, Gly206, Arg207, Asp210 and Ser211, are marked in cyan frames and shown in space-filling representation. The Na^+^ ion coordinated in the stabilization loop is shown as a purple sphere.

The prototypic class 2 enzyme is EcAIII from *E. coli*. EcAIII belongs to the Ntn-hydrolases, which are expressed as immature polypeptides (Borek *et al.*, 2004 ▸) that dimerize and then autocatalytically cleave themselves into α (∼20 kDa) and β (∼15 kDa) subunits to form the mature (αβ)_2_ heterotetramer (a dimer of two αβ heterodimers; Fig. 1 ▸). In the precursor protein, the uncleaved α and β polypeptides form two structural domains connected by a linker (Fig. 1 ▸). After cleavage, the nucleophilic residue (Thr179 in EcAIII numbering) is liberated at the free N-terminus of the β subunit. The same Thr179 acts as the nucleophile in the autoproteolytic maturation and in l-asparagine hydrolysis.

Autocleavage is initiated by nucleophilic attack of the side chain of Thr179 on the carbonyl C atom of the peptide bond (Gly178-Thr179) preceding it (Li *et al.*, 2012 ▸). Next, a tetrahedral transition state is formed and stabilized by the oxy­anion hole created by the side chain of Asn67 and a water molecule. In the final step, a nucleophilic water molecule hydrolyzes the tetrahedral transition state to liberate Thr179 at the N-terminus of the β subunit (Michalska *et al.*, 2008 ▸).

The l-asparagine hydrolysis reaction is most likely to proceed via a ping-pong mechanism (Aghaiypour *et al.*, 2001 ▸). It is initiated by nucleophilic attack of Thr179 on the C^γ^ atom of the substrate. In the first displacement event, the negatively charged tetrahedral transition state is stabilized by the oxy­anion hole built by Thr230 (hydroxyl group) and Gly231 (NH group). This oxyanion hole differs from that utilized in the maturation process (Michalska *et al.*, 2005 ▸). Collapse of the tetrahedral transition state releases ammonia and leaves the substrate covalently attached to Thr179. In the second displacement step, the covalent intermediate is hydrolyzed and another tetrahedral transition state appears. The reaction is completed by release of the l-Asp product (Nomme *et al.*, 2012 ▸).

**Figure 2:**
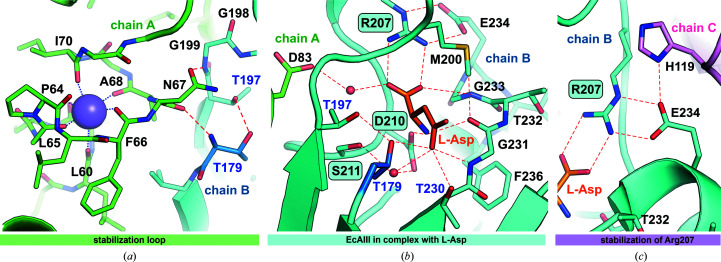
Interatomic interactions in the stabilization loop and the active site of EcAIII. (*a*) Detailed view of the stabilization loop; the octahedral coordination of the sodium cation (purple sphere) is marked by dotted blue lines. (*b*) Residues involved in substrate/product (orange) binding in the active site (PDB entry 2zal). The oxyanion hole is formed by Gly231 (NH group) and Thr230 (OH group). (*c*) Stabilization of Arg207 by Glu234 and His119 in its substrate-binding position. Residues subjected to random mutagenesis are marked in cyan frames. The conserved threonine triad is comprised of Thr179 (dark blue), Thr197 and Thr230. The α subunit (chain *A*) is colored green, the β subunit (chain *B*) is colored cyan and the α subunit from the complementary dimer (chain *C*) is colored light pink. Hydrogen bonds are marked as red dashed lines and water molecules as red spheres.

The active site of EcAIII is located in subunit β, between a sandwich of two β-sheets flanked on each side by a layer of α-helices (Fig. 1 ▸). In addition to the nucleophilic Thr179, it includes two other threonines forming a threonine triad: Thr179–Thr197–Thr230 (Fig. 2 ▸). Other residues participating in substrate/product binding are located in the close vicinity (Fig. 2 ▸). Arg207 and Gly233 are responsible for the anchoring of the α-carboxyl group, while Thr230 and Thr179 stabilize the γ-carboxyl group of the substrate or product. Asp210 and Gly231 are hydrogen-bonded to the α-amino group of the substrate or product. Ser211 and Asp83 participate in binding of α-amino group of the substrate or product via water-mediated hydrogen bonds. Another structural element that is important for supporting the hydrogen-bond pattern in the active site is a sodium-binding loop (stabilization loop) comprised of residues Phe66–Ile70 (Fig. 2 ▸).

The unique architecture and properties of class 2 l-asparaginases (compared with class 1) make them interesting candidates for novel therapeutics, with a potentially tunable self-activation process and controlled enzymatic activity. Moreover, class 2 enzymes have a very low (Sun *et al.*, 2016 ▸) or undetectable (Cantor *et al.*, 2009 ▸) glutaminase side activity, which may be very beneficial in reduction of the therapeutic side effects (Fonseca *et al.*, 2021 ▸). Unfortunately, native class 2 l-asparaginases have an affinity for l-Asn that is too low (millimolar) for their potential utilization in medicine. While enzyme engineering offers excellent opportunities to improve substrate affinity, it can only be efficient if the mechanism of action of the enzyme is known in detail. This work reports the results of random mutagenesis of the model *E. coli* enzyme EcAIII. Our experiments were aimed at analyzing the influence of different mutations on the activity and stability of EcAIII. Unexpectedly, randomization of the selected sites resulted in the generation of catalytically inactive mutants that differed, however, in their autoprocessing ability. Therefore, we carried out a detailed structural and biochemical characterization of selected variants to identify the structural factors that determine the ability of EcAIII to undergo auto­proteolytic maturation. The conclusions from our work, discussed in the context of the properties of class 2 l-aspara­ginases and other Ntn-hydrolases and grouped into six lessons, provide useful hints that can be applied to future fine-tuned engineering of EcAIII and also of other Ntn-amidohydrolases, as most class 2 enzymes have a highly conserved active-site architecture.

## 2. Methods

### 2.1. Random mutagenesis, monitoring of clone activity and autoprocessing

Random mutagenesis of the EcAIII sequence was performed with the use of degenerate primers, designated ‘RDM1’ (to denote our first campaign of random mutagenesis; Supplementary Fig. S1), introducing random amino acids at positions 206, 207, 210 and 211 of the protein sequence. In this naming scheme, all mutants described in this work are labeled ‘RDM1-*X*’, where *X* is the ordinal number of a particular bacterial colony that was screened (Table 1 ▸, Supplementary Fig. S2). PCR was performed according to the classical QuikChange protocol. As a PCR matrix, the EcAIII gene cloned into the pET-11d vector was used. After PCR, the matrix was digested with DpnI ribonuclease and the PCR products were transformed into *E. coli* BL21 Gold cells.

Following overnight incubation on plates supplemented with 100 µg ml^−1^ ampicillin, single colonies were randomly selected and transferred into 1 ml liquid LB medium. After overnight growth, 0.5 ml liquid cultures were added to 4 ml fresh LB medium with ampicillin and were vigorously agitated at 37°C for 4 h. Next, the cultures were incubated for 30 min at 4°C, after which time protein expression was induced with 2 m*M* isopropyl β-d-1-thiogalactopyranoside (IPTG) and the cultures were incubated at 18°C with shaking. After overnight incubation, 0.5 ml of each culture was centrifuged. The pellet was resuspended in 90 µl 50 m*M* Tris–HCl pH 8.0 buffer, followed by the addition of 10 µl of the lysis reagent CelLytic B (Merck). Cell lysis was performed for 15 min at room temperature with vigorous shaking. The cell suspension was centrifuged and the clarified cell lysate was subjected to an l-asparaginase activity test.

l-Asparaginase activity was monitored at pH 8.0 (50 m*M* Tris–HCl) in 96-well plates using Nessler’s reagent and 10 m*M* l-Asn as a substrate. After a 15 min incubation of 10 µl cell lysate with substrate, 30 µl Nessler’s reagent was added and the development of color was observed. All experiments were performed together with a positive control, which consisted of a cell lysate containing wild-type (WT) EcAIII expressed in parallel under the same conditions as the analyzed clones, and a negative control, which consisted of a cell lysate of untransformed *E. coli* BL21 Gold cells incubated under the same conditions. The level of expression of randomized EcAIII (as well as of WT EcAIII) and the autoprocessing into submits α/β was monitored by SDS–PAGE. After activity tests and analysis of the SDS–PAGE gels, selected plasmids encoding randomized protein sequences were isolated and sequenced (Genomed, Poland). Successfully sequenced clones were subjected to large-scale protein expression and biophysical studies.

### 2.2. Large-scale protein expression and purification

Starting cultures were transferred into 1 l LB medium and the cells were grown at 37°C until the OD_600_ was close to 1.00. Next, the cultures were cooled to 4°C for 30 min and protein expression was induced using 0.5 m*M* IPTG. Expression was carried out overnight at 18°C. The cells were harvested by centrifugation. The cell pellets were resuspended in 20 m*M* Tris–HCl pH 8.0 buffer and cell lysis was performed by sonication followed by centrifugation. The clarified cell lysate was fractionated by ion-exchange chromatography (DEAE-Sepharose, Merck). Fractions were eluted from the column using a gradient of 2 *M* NaCl in 20 m*M* Tris–HCl pH 8.0. Fractions were analyzed by SDS–PAGE and those containing the desired protein were pooled, concentrated and loaded onto a size-exclusion chromatography (SEC) column (Sephadex G75, GE Healthcare) connected to an ÄKTA FPLC chromatography system (GE Healthcare).

### 2.3. Biophysical measurements

Before biophysical measurements, the proteins were additionally purified by another SEC step on Superdex 75 10/300 GL (GE Healthcare) using 20 m*M* phosphate buffer pH 7.5. Far-UV CD spectra were recorded on a Jasco J-815 spectropolarimeter using a protein concentration range of 0.5–1.0 mg ml^−1^ and a 1 mm path length. Spectra were corrected for buffer baseline and were normalized to the same value at 222 nm. Thermal stability was monitored by nanoDSF using a Prometheus NT.48 (NanoTemper Technologies) and a sample volume of ∼10 µl per capillary (protein concentration 0.5–1.0 mg ml^−1^). An initial florescence scan (emission at 330 and 350 nm) showed that all samples were within the detectable concentration range. Melting scans were recorded as fluorescence emission for protein samples subjected to a 20–95°C temperature ramp at 1°C min^−1^.

### 2.4. Crystallization, X-ray data collection and structure refinement

Proteins (in the concentration range 10–15 mg ml^−1^) were crystallized by vapor diffusion in hanging drops at 20°C. Crystals were grown from solutions consisting of 20–30% PEG 4000 or PEG 6000, 0.2 *M* MgCl_2_ in 100 m*M* Tris–HCl pH 8.5. X-ray diffraction data were collected using synchrotron radiation on the EMBL beamlines at the PETRA III storage ring at DESY in Hamburg or using Cu *K*α radiation from a home-source Synergy-S or Synergy-R XtaLAB (Rigaku) generator. The diffraction images were processed and scaled using the *CrysAlis^Pro^* (Rigaku), C*CP*4 (Winn *et al.*, 2011 ▸) or *XDS* (Kabsch, 2010 ▸) packages. Structures were solved by molecular replacement using *Phaser* (McCoy *et al.*, 2007 ▸) and were refined with *REFMAC*5 (Murshudov *et al.*, 2011 ▸) using anisotropic or TLS protocols. The electron-density maps were inspected in *Coot* (Emsley *et al.*, 2010 ▸). All crystal structures were standardized in the unit cell using the *ACHESYM* server (Kowiel *et al.*, 2014 ▸). All data-collection and structure-refinement statistics are summarized in Table 2 ▸. The structures were analyzed and visualized using *PyMOL*. Together with the series of crystal structures of the RDM1 mutants, the structure of the WT protein (without bound ligand) was determined at 1.55 Å resolution.

### 2.5. Bioinformatic analyses

The three-dimensional structure of the immature precursors possessing an intact linker was predicted from the amino-acid sequences of the WT and all other new variants of EcAIII using the *AlphaFold*2 code (Jumper *et al.*, 2021 ▸). The artificial intelligence (AI) modeling was performed for a single WT EcAIII protomer and, for reference, for the dimeric structure. However, due to imperfections in the *AlphaFold*2 algorithm in application to oligomeric proteins, clashes between residues were frequently observed in the latter case. Therefore, eventually these results were rejected and the analysis was limited to the ‘monomeric’ structures of the unprocessed variants.

## 3. Results

### 3.1. Mutagenesis

In the mutagenesis experiment, residues at four positions were randomized (Table 1 ▸). The mutation sites were selected based on an analysis of the crystal structure of WT EcAIII as follows. Gly206 precedes Arg207, which is crucial for the binding of the α-carboxylate group of the substrate, while Ser210 is located in front of Asp211, which is responsible for the binding of the amino group of the l-Asn substrate (Fig. 2 ▸). Because of their involvement in substrate binding, these residues (*i.e.* Arg207 and Asp211) are necessary for the l-asparaginase activity. However, their contribution to the autocleavage process is at present unknown.

In our studies, a total of 64 randomized clones were analyzed, *i.e.* subjected to l-asparaginase activity (Nessler’s reaction) and autoproteolytic (SDS–PAGE) tests (Supplementary Fig. S2). The SDS–PAGE analysis showed that almost all of the variants were efficiently produced on a small scale with similar expression levels. The Nessler’s test revealed that all mutants were unable to hydrolyze l-Asn. The SDS–PAGE gels revealed that most of the variants remained in the unprocessed, immature form (no bands corresponding to α/β subunits). However, autoprocessing was clearly visible for some clones. In some cases, a single band with an atypical molecular weight on the gel suggested that expression of the full-length protein was aborted by a STOP codon introduced into the sequence (Supplementary Fig. S2). To check the origins of the lack of autoprocessing, randomly selected genes encoding 21 clones (12 proteins that were processed into subunits and nine that were unprocessed) were subjected to DNA sequencing (Supplementary Fig. S2). Finally, 15 protein genes were analyzed (eight processed and seven unprocessed proteins). The sequence-analysis results are summarized in Table 1 ▸.

### 3.2. Thermal stability and CD spectroscopy

**Figure 3:**
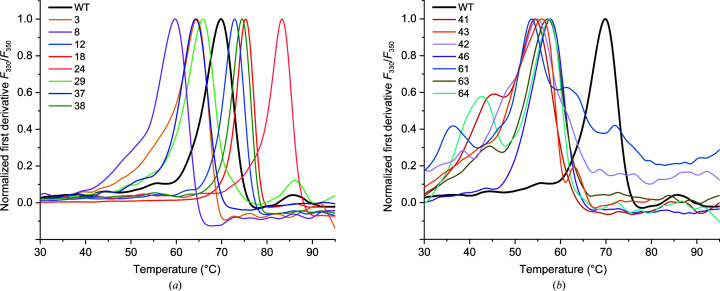
Melting profiles of the new EcAIII variants. The variants are marked by color according to their number in Table 1 ▸. (*a*) Mutants processed into subunits α/β; (*b*) mutants incapable of autoprocessing.

Most of the RDM1 mutants retained the secondary (Supplementary Fig. S3) and quaternary structure of the WT protein (Supplementary Fig. S4). Normalized melting curves of the analyzed variants are presented in Fig. 3 ▸, while the *T*_m_ values are summarized in Table 1 ▸. The results indicated that all of the unprocessed variants had a thermal stability that was decreased by more than 10°C compared with the WT protein. Moreover, a multi-step denaturation process was observed for most of them. Analysis of *T*_m_ of the autoprocessing mutants reveled that they can be divided into two groups: those with decreased (RDM1-3, RDM1-8, RDM1-29 and RDM1-37) and increased (RDM1-12, RDM1-18, RDM1-24 and RDM1-38) *T*_m_ values (Fig. 3 ▸, Table 1 ▸). All of them have the same melting profile, with a single transition at the given *T*_m_. Prior to crystallization experiments, the protein fold was analyzed by CD spectroscopy (Supplementary Fig. S3). In general, the spectra for almost all mutants are similar to that of the WT protein; however, for all unprocessed variants a decrease in signal intensity at 195 nm was observed, indicating systematic structural distortions of these variants, which was probably caused by dynamic disorder of the linker.

### 3.3. Crystal structures and conformational changes caused by the mutations

It was possible to crystallize all of the autoprocessing mutants, while extensive screening for crystallization conditions did not produce any crystals of the immature variants. The quality of the crystals of the mutants processed into subunits varied, indicating that the mutations also affected the crystallization process. Although all crystals have very similar morphology, they diffracted X-rays to different degrees. As a result, the crystal structures were determined with a maximum resolution in the range 1.20–2.55 Å. The refinement statistics in Table 2 ▸ reflect the quality of the diffraction data. The isomorphism of WT EcAIII and the mature variants (space group *P*2_1_2_1_2_1_) greatly facilitated comparisons of the structures. In general, analysis of the structures revealed that substitutions at positions 210 and 211, located in geometrical proximity of the nucleophilic Thr179, did not affect the position or conformation of this residue, and the canonical pattern of hydrogen bonds within the threonine triad remained unchanged.

More significant changes were visible in the region of substitutions at positions 206 and 207. In the WT protein, the side chain of Arg207 is responsible for presenting the substrate molecule to the nucleophilic warhead and for maintaining the pattern of hydrogen-bond connections in the active site (Fig. 2 ▸). As it was replaced by residues with other side chains (*e.g.* Thr, Ala, Cys, Gly, Ser or Val; Table 1 ▸), these changes explain the lack of l-asparaginase activity of the corresponding variants, as none of the newly inserted residues has the ability to bind the α-carboxylate group of the substrate and thus to properly orient the l-Asn substrate for the hydrolytic reaction.

**Figure 4:**
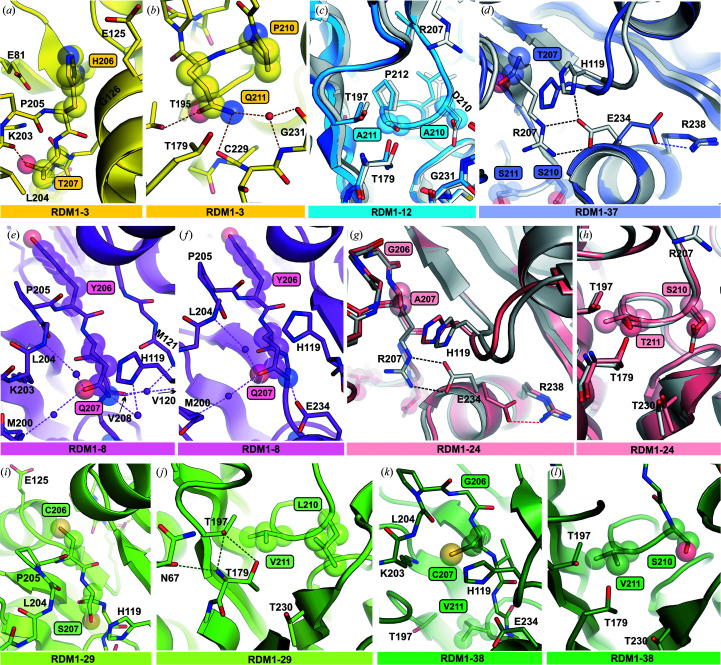
Structural changes in mutants from the RDM1 series: RDM1-3 (yellow), RDM1-12 (blue), RDM1-37 (violet), RDM1-8 (magenta), RDM1-24 (salmon), RDM1-29 (light green) and RDM1-38 (dark green). The WT protein, shown for reference, is colored light gray. The mutation sites are marked in frames. Hydrogen bonds are shown as dashed lines and water molecules as spheres. For clarity of viewing, some side chains were omitted in the figures. (*a*) RDM1-3; Thr207 is hydrogen-bonded to Leu204, while His206 occupies the free space between Glu125 and Glu81. (*b*) RDM1-3; Gln211 is involved in an extended network of hydrogen bonds, while Pro210 is neutral for the conformation of the neighboring residues. (*c*) RDM1-12; two nonpolar substitutions at positions 210 (D/A) and 211 (S/A) do not affect the conformation of the adjacent side chains. (*d*) RDM1-37; a shift of His119 induces a swing of the Glu234 side chain, forcing it form a hydrogen bond to Arg238; in the WT protein Glu234 is hydrogen-bonded to Arg207 and His119. (*e*) RDM1-8 (chain *B*); Gln207 forms several water-mediated hydrogen bonds to protein main-chain atoms. (*f*) RDM1-8 (chain *D*); Gln207 is hydrogen-bonded to Glu234, which has a different orientation than in chain *B*. (*g*) RDM1-24; the substitution R207A is neutral for the conformation of the neighboring side chains. (*h*) RDM1-24; substitutions D210S and S211T do not affect the position of the catalytic Thr179. (*I*) RDM1-29; Cys206 is directed away from the active site. (*j*) RDM1-29; two nonpolar substitutions, S211V and D210L, in the close neighborhood of Thr179. (*k*) RDM1-38; position and orientation of Cys207 in chain *B*. (*l*) RDM1-38; nonpolar substitution of S211V in the close vicinity of the catalytic Thr179.

In addition to switching off the l-asparaginase activity, the absence of Arg207 caused other structural rearrangements. Specifically, in most of the variants the side chain of His119 was shifted by about 1.5–2.2 Å into the center of the active-site area, thus blocking the catalytic center, while Glu234, which normally forms an anchoring salt bridge with Arg207, rotated by ∼180° to form a similar salt bridge with Arg238 (Fig. 4 ▸ *d*). These changes also affected the position of the entire loop 200–208 and the region located in front of it. As each of the discussed variants has an individual pattern of substitutions, the details of the structural changes are described separately in the following subsections.

#### 3.3.1. Variants RDM1-3, RDM1-12 and RDM1-37 (all with the R207T mutation)

Variant RDM1-3, in addition to the R207T substitution, also carries a G206H mutation. The side chain of His206 is exposed outside the active site and occupies the space between Glu81, Glu125 and Gly126. Thr207 is hydrogen-bonded to the carbonyl O atom of Pro204, and this interaction stabilizes the entire 200–208 loop (Fig. 4 ▸ *a*). Two other substitutions, D210P and S211Q, located closer to the nucleophilic Thr179, do not affect the conformation of the neighboring residues. The relatively long side chain of Gln211 fits well into a cleft surrounded by Thr179, Thr230 and Pro210, and forms a hydrogen bond to the O^γ1^ atom of Thr195 and the carbonyl O atom of Cys229 (Fig. 4 ▸ *b*).

In mutant RDM1-12, Cys206 and Thr207 do not affect the neighboring residues. The D210A and S211A substitutions, located close to the threonine triad, despite the presence of two small nonpolar alanines (Fig. 4 ▸ *c*), do not affect the conformation of the adjacent side chains but modify the pattern of hydrogen bonds to water molecules. The side chain of Glu234, rotated towards Arg238, has a slightly different conformation and a different pattern of hydrogen bonds in the two subunits.

Mutant RDM1-37 has a serine instead of Asp at position 210. Its O^γ^ atom is hydrogen-bonded via two water molecules to the side chain of Ser211, which was not replaced in this variant. The side chain of Glu234 is rotated towards Arg238 (Fig. 4 ▸ *d*); its conformation is different in each dimer subunit but is very similar to the conformation observed in variant RDM1-12.

#### 3.3.2. Variants RDM1-8 and RDM1-24 (all with the S211T mutation)

Variant RDM1-8 has two relatively large substitutions at positions 206 (Tyr) and 207 (Gln). The side chain of Tyr206 occupies the space between Glu81 and Glu125. The side chain of Gln207 is directed towards the active site, and in one dimer subunit (*B*) is hydrogen-bonded via water molecules to the N atoms of Leu204, Met200, Val120 and Met121, the carbonyl O atom of Val208 and the side chain (N^δ1^) of His119 (Fig. 4 ▸ *e*). The nearby side chain of Glu234 is rotated towards Arg238. In contrast, in the second dimer subunit (*D*) Glu234 is directed towards the center of the active site and is hydrogen-bonded to the N^ɛ2^ atom of Gln207 (Fig. 4 ▸ *f*). Gln207 also forms water-mediated hydrogen bonds to the side chain of His119 and to the N atoms of Leu204 and Met200 (Fig. 4 ▸ *f*).

In variant RDM1-24, Arg207 was replaced by Ala (Fig. 4 ▸ *g*). The small side chain of Ala at position 207 is neutral; it does not affect the protein structure and does not participate in hydrogen bonds. In both the RDM1-8 and RDM1-24 variants the D210S/S211T substitutions are similar in their chemical character (Fig. 4 ▸ *h*) to the natural residues and support the pattern of hydrogen bonds typical of the WT protein.

#### 3.3.3. Variants RDM1-29 and RDM1-38 (all with the S211V mutation)

In variant RDM1-29, the side chain of Cys206 is directed towards Glu125 (Fig. 4 ▸ *i*). Despite the presence of two nonpolar substitutions, D210L and S211V (Fig. 4 ▸ *i*), in the close vicinity of Thr179, mutant RDM1-29 is efficiently cleaved into α/β subunits. Similar observations were made for variant RDM1-12 with Ala at positions 210 and 211.

In variant RDM1-38, Arg207 was replaced by Cys (Fig. 4 ▸ *k*). The introduction of Cys207 resulted in small rearrangements in the Lys203–Val208 region. The side chain of Cys207 has a slightly different orientation in the two dimer subunits. Glu234 has two conformations in both subunits: one with its side chain directed towards Arg238 and another with it directed towards His119. At position 211 a nonpolar Val is present, but this substitution does not affect the conformation of the catalytic Thr179 or the efficiency of the autocleavage process (Fig. 4 ▸ *l*).

#### 3.3.4. Variant RDM1-18

**Figure 5:**
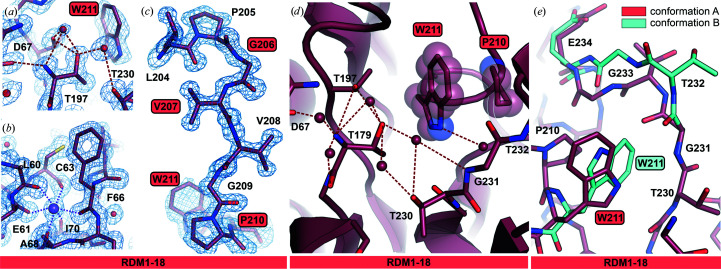
Structure of mutant RDM1-18 determined at 1.20 Å resolution. 2*F*_o_ − *F*_c_ electron-density map (contour level 1.50σ) near (*a*) the catalytic Thr179 and (*b*) the sodium-binding loop in chain *B*; the sodium cation is colored violet. (*c*) 2*F*_o_ − *F*_c_ electron-density map (contour level 1.50σ) around the mutation sites in chain *B*. (*d*) Conformation of Trp211 in chain *B*; the presence of the bulky aromatic side chain does not affect the pattern of hydrogen bonds near the Thr triad. (*e*) Alternative conformations of Trp211 and the neighboring fragment Thr232–Glu234 in chain *D*. In all panels, the mutation sites are marked in frames. Water molecules are shown as red spheres and hydrogen bonds as dashed lines.

Crystals of variant RDM1-18 diffracted X-rays very strongly, allowing data collection to 1.20 Å resolution (Table 2 ▸). As this is currently the highest resolution EcAIII structure deposited in the PDB, this variant will be discussed separately. High-quality electron-density maps allowed us to observed fine details, for example the environment of Thr179 and the octahedral coordination sphere of the Na^+^ ion in the stabilization loop (Figs. 5 ▸ *a*–5 ▸ *c*). In mutant RDM1-18 the D211W substitution introduced a bulky Trp side chain into the immediate neighborhood of the Thr179 nucleophile. Nevertheless, this mutation did not affect autoprocessing. The side chain of Trp211 filled the available space between the threonine triad and the fragment built from residues 207–210 containing the S211P substitution (Fig. 5 ▸ *d*). In chain *B*, disorder was detected in the active site, while in the corresponding region of subunit *D* two alternative conformations of Trp211 are visible. The double conformation of Trp211 is correlated with the behavior of the 230–236 fragment, for which two alternative conformations are also visible (Fig. 5 ▸ *e*).

### 3.4. Autocleavage and conserved water molecules

It has been reported that at least two water molecules are crucial for the autocatalytic reaction: one (w1) that participates in the creation of the oxyanion hole that stabilizes the transition state during autocleavage and another one that hydrolyzes the covalent ester intermediate (Su *et al.*, 2013 ▸). Water w1 also enhances the nucleophilicity of the catalytic Thr residue, but its exact location might vary in different Ntn-hydrolases (Okada *et al.*, 2007 ▸; Yoon *et al.*, 2004 ▸). While w1 was identified in the crystal structure of the T179A mutant of EcAIII (Michalska *et al.*, 2008 ▸), the second water molecule has not been found to date. Our exercise to identify conserved water molecules was carried out via C^α^ superposition of all RDM1 structures.

**Figure 6:**
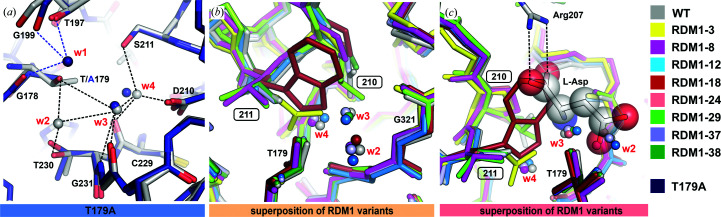
Conserved water molecules in EcAIII structures. (*a*) Superposition of WT EcAIII and the T179A mutant (PDB entry 3c17); water molecule w1 is part of the oxyanion hole formed during autoprocessing, while waters w2, w3 and w4 are conserved among almost all variants from the RDM1 series. (*b*) Superposition of all of the structures determined in this work showing the positions of waters w2, w3 and w4. (*c*) Superposition of mutants from the present RDM1 series and the complex of EcAIII with l-Asp (PDB entry 2zal); water molecules w2 and w3 superpose with l-Asp, while Trp211 in RDM1-18 occupies the space needed for substrate/product binding (shown in sphere representation). The protein coloring scheme is presented on the right. In all panels, the mutation sites are marked in frames. Water molecules are shown as spheres and hydrogen bonds as dashed lines.

The analysis revealed that water 1 is absent in all variants. Water 1 should be hydrogen-bonded to the carbonyl O atoms of Gly178 and Gly199 and to O^γ1^ of Thr197 (Fig. 6 ▸); however, the fragment covering residues 158–178 is not visible in the structures of the RDM1 series. In most of the structures there is a water molecule w2, which is hydrogen-bonded to the O^γ1^ atoms of Thr179 and Thr230, water w3, which is hydrogen-bonded to w2 and the N and O atoms of Gly231, and another water w4, which is coordinated by the side chains of residues at position 210 and Ser211 (when possible) (Figs. 6 ▸ *a* and 6 ▸ *b*). Waters w3 and w4 are absent in variants with bulky side chains at position 211, such as Gln (RDM1-3) or Trp (RDM1-18). The positions of other water molecules identified in the RDM1 mutants are variable, but most of these waters superpose well on the product molecule bound in the active site (PDB entry 2zal; Fig. 6 ▸ *c*). This observation indicates that waters w2, w3 and w4 are coordinated in the empty active site instead of the substrate. Moreover, they are located too far from Thr179 to participate in autoproteolysis.

Interestingly, despite the presence of substitutions 210 and 211, in most variants (except RDM1-18) there is still room for substrate binding; however, the absence of the Arg207 side chain abrogates anchoring of l-Asn/l-Asp in the active site. The above analysis indicates that there is no conserved system of hydrogen bonds involving water molecules close to the nucleophilic Thr179 that can potentially be engaged in the autocleavage event.

### 3.5. Prediction of the structures of the immature precursors

Earlier reports suggested that a highly strained conformation of the peptide bond between Gly178 and Thr179 is the driving force behind the autocleavage (Li *et al.*, 2012 ▸). As the crystal structure of the uncleaved WT precursor protein with full definition of the linker is not currently available, insight into the autocleavage event may be gained from predicted models. We carried out such modeling using the *AlphaFold*2 AI algorithm (Jumper *et al.*, 2021 ▸) to obtain 3D models of the immature precursors of the WT protein (serving as a reference) and variants from the RDM1 series that were not cleaved into subunits.

**Figure 7:**
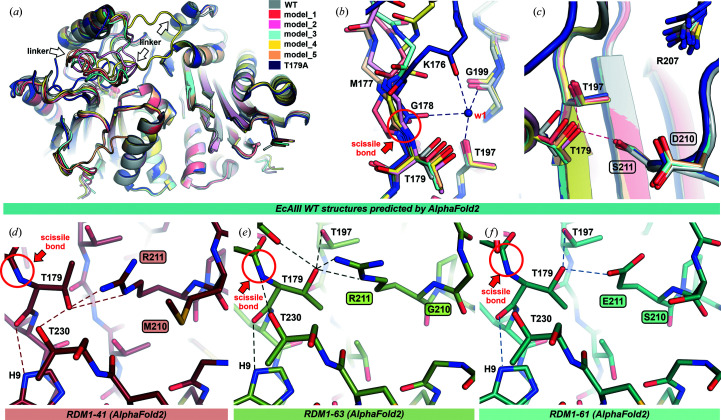
Structures of WT EcAIII and its variants predicted by *AlphaFold*2. (*a*) Superposition of WT EcAIII and its five best models predicted by *AlphaFold*2 (color legend in inset); although all core structures were predicted correctly, the conformation of the linker is different in each model. (*b*) Position of Thr179 in the predicted models of WT EcAIII superposed on the crystal structure of the T179A mutant (PDB entry 3c17); the lack of water molecules in the predicted models might lead to incorrect conformations of the side chain of Thr179 and of the scissile bond. (*c*) In some models, the distance between Thr179 and Ser211 is close enough to suggest hydrogen bonding. (*d*) and (*e*) show the predicted hydrogen bonds between Arg211 and Thr179 in variants RDM1-41 and RDM1-63. (*f*) Mutant RDM1-61 with a hydrogen bond between Glu211 and Thr179. In all panels, the mutation sites are marked in frames and hydrogen bonds are marked as dashed lines.

The modeled structures of EcAIII (the five best predictions) superimposed well with the crystal structure of the WT protein, with r.m.s.d.s of ∼0.44 Å for C^α^ atoms and ∼1.00 Å for all non-H atoms. The predictions, therefore, can be treated with confidence. For linker region (residues 156–178) slightly different conformations were observed (Fig. 7 ▸ *a*). As a consequence, the position of the fragment including the scissile bond Gly178-Thr179 also varied, suggesting that flexibility of the linker might be an important factor affecting the efficiency of autocleavage, influencing the degree of optimal presentation of the scissile bond for nucleophilic attack. Moreover, superposition of the predicted models and the uncleaved T179A mutant (PDB entry 3c17) shows that water 1 is necessary to stabilize the scissile bond in the proper orientation for cleavage. The lack of water molecules in the predicted structures may mean that these models do not reflect physical reality and are thus of limited use in comparative analysis.

Despite these limitations, we used *AlphaFold*2 to model the mutants that were not cleaved into subunits (Supplementary Fig. S5). In each run of AI modeling, five models were produced for each uncleaved variant. The models varied in the position of the linker, while the positions and conformations of residues in the active site and in the region of the scissile bond were preserved. In Fig. 7 ▸ and Supplementary Fig. S5 only one representative prediction is presented for each case. In the case of variants RDM1-41, RDM1-61 and RDM1-63 the predicted structures gave a clear answer as to why autoprocessing was not possible. Mutants 63 and 41 possess Arg in position 211. This Arg residue forms two hydrogen bonds to the O^γ^ atom of the Thr179 nucleophile, thereby arresting its movement and preventing its attack on the scissile bond preceding it. Even more importantly, as a positively charged hydrogen-bond donor, arginine drastically reduces the nucleophilic character of Thr179. Additionally, in the case of variant RDM1-41 the side chain of Thr179 is rotated into a new position that is incompatible with a nucleophilic attack on the scissile bond Gly178-Thr179 (Fig. 7 ▸ *d*). Mutant RDM1-61 has Glu at position 211, which is also hydrogen-bonded to the O^γ^ atom of Thr179. Although the negatively charged Glu can potentially enhance the nucleophilic character of Thr179 and thus boost autoprocessing, such an effect was not observed.

In the modeled precursors of the remaining variants, RDM1-42, RDM1-43, RDM1-46 and RDM1-64, no specific interactions between the mutated residues and Thr179 were found (Supplementary Fig. S5) and the models did not explain why their autoprocessing is abrogated. Of particular interest is the case of RDM1-43, which has the same D210S substitution as mutant RDM1-37, and yet in contrast to the latter variant lost its ability to autocleave (Supplementary Fig. S6). In both RDM1-43 and RDM1-37 Ser at position 211 was preserved (Table 1 ▸). Mutants RDM1-37 and RDM1-43 differ in the type of substitutions at positions 206 and 207 (206→S/V and 207→T/G, respectively). It is possible that these two differences changed the mobility (conformational disorder) of the linker and significantly lowered the probability of proper conformation and orientation at the scissile bond.

## 4. Discussion

In this work, random mutagenesis of the prototypic class 2 l-asparaginase from *E. coli*, EcAIII, was performed, with the caveat that the analysis of clones was carried out manually for a relatively small set of 15 clones. The current approach to enzyme modification is directed evolution; however, it requires a high-throughput selection system. To date, directed evolution has been applied to the following l-asparaginases: (i) the class 1 enzyme from *B. megaterium* (Lu *et al.*, 2019 ▸), (ii) the class 1 enzyme from *E. chrysamthemi* (Kotzia & Labrou, 2009 ▸), (iii) chimeric human/guinea pig enzymes (class 1; Rigouin *et al.*, 2017 ▸) and (iv) human Ntn-hydrolase HsAIII (class 2; Karamitros & Konrad, 2016 ▸). The selection system for (i) and (ii) was based on activity screening directly in *E. coli* BL21(DE3) lysates and that for (iii) on observation of *E. coli* (strains BW2Δ/BW5Δ) colony growth, while for (iv) it combined several approaches [expression in *E. coli* strain C41(DE3), purification, observation of JC1(DE3) colonies and fluorescence monitoring]. All of these selection systems were based on prokaryotic expression hosts.

As reported previously, only Ntn-hydrolases of bacterial (Michalska *et al.*, 2005 ▸) and plant (Bejger *et al.*, 2014 ▸; Borek *et al.*, 2004 ▸) origin can efficiently be cleaved into subunits in *E. coli* cells, while mammalian enzymes require purification and glycine-accelerated maturation (Schalk & Lavie, 2014 ▸; Su *et al.*, 2013 ▸). For this reason, we decided to use the prokaryotic EcAIII to monitor the influence of mutations on class 2 enzyme activity. Furthermore, it was reported previously that whole-gene randomization of human Ntn-hydrolase HsAIII was inefficient, leading to incomplete libraries (Karamitros & Konrad, 2016 ▸). Therefore, we decided to use local randomization of four active-site residues of EcAIII to increase the probability of obtaining highly diversified libraries. The observations originating from our randomization of the EcAIII active site, as well as their implications (*i.e.* lessons) for future experiments, are discussed in detail in separate sections.

### 4.1. Lesson 1: the entire EcAIII fold adapts well to new substitutions

The random mutagenesis of the EcAIII protein presented in this work is the first attempt to investigate how well the prototypic Ntn-hydrolase can tolerate multiple simultaneous substitutions in the active site. Some single site-directed mutagenesis experiments have been performed previously for similar enzymes to elucidate the catalytic and autoproteolytic mechanisms (Nomme *et al.*, 2014 ▸; Michalska *et al.*, 2008 ▸; Ajewole *et al.*, 2018 ▸). Our sequencing analysis showed that the frequencies of residues at selected mutation sites are not uniform, but this is a typical result of local randomization with the use of degenerate codons (Tang *et al.*, 2012 ▸).

Among bacterial class 2 l-asparaginases, the residues (EcAIII numbering) Gly206, Arg207, Asp201 and Ser211 are highly conserved. At position 211, in addition to the most frequent Ser, Thr or Ala can also be found (Zielezinski *et al.*, 2022 ▸). Our studies demonstrate that even the highly conserved regions of EcAIII can be modified, although some perturbations of protein folding were observed for variants that were not cleaved into subunits (for example RDM1-42), as indicated by the CD spectra (Supplementary Fig. S3). A variety of substitutions that were introduced in the active-site region of the fully processed EcAIII variants, polar (Thr, Gln, Ser), nonpolar (Ala, Val, Cys, Leu, Pro) or aromatic (Tyr, His) residues, showed that the structure of EcAIII accommodates multiple mutations very well. Despite local conformational changes and atomic shifts, all mutants were produced in soluble form and most retained the correct secondary structure and heat stability above 50°C.

Thermal stability is an important factor to consider when designing proteins for medicinal applications. An optimal *T*_m_ is required for manufacturing as well as transport, storage and administration of enzymes (Krause & Sahin, 2019 ▸; Bansal *et al.*, 2012 ▸, 2021 ▸; Asial *et al.*, 2013 ▸; Modarres *et al.*, 2016 ▸). As a rule, precursors of Ntn-hydrolases have a highly strained conformation at the scissile peptide bond (Wang & Guo, 2003 ▸), which is manifested by a lower thermal stability of the entire protein. The *T*_m_ values determined for the RDM1 variants that were not cleaved into subunits were 11–15°C lower relative to that of the WT protein. These observations are consistent with the data reported by Li *et al.* (2016 ▸) for HsAIII. The unprocessed precursor of HsAIII had a *T*_m_ of ∼61°C, while full autoprocessing increased its thermal stability to ∼70°C. In the case of the EcAIII variants that were processed into subunits, we can observe both an increase and a decrease in *T*_m_ depending on the variant.

**Figure 8:**
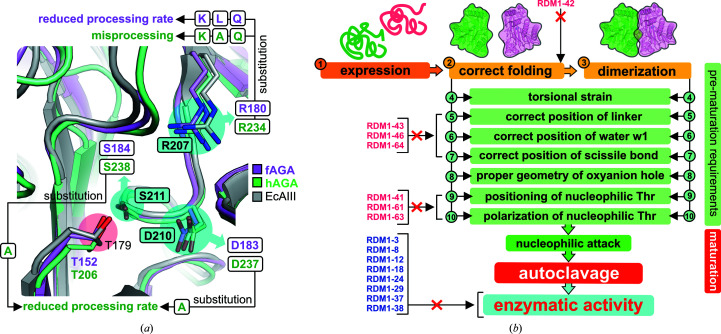
(*a*) Superposition of EcAIII (gray) and structural homologs, *F. meningosepticum* AGA (fAGA, pink) and human AGA (hAGA, green), with the counterparts of selected EcAIII residues shown as sticks: Arg207 (Arg180/234 in fAGA/hAGA), Asp210 (Asp183/237 in fAGA/hAGA) and Ser211 (Ser184/238 in fAGA/hAGA). Black arrows mark the type of point mutations in fAGA (in magenta) and hAGA (in green) that led to misprocessing or reduced processing of AGA precursors. The cyan patches and frames mark the EcAIII sites mutated in this work and the pink patch marks the nucleophilic Thr residue. (*b*) Structural requirements necessary to initiate the maturation of Ntn-amidohydrolases and classification of the RDM1 variants of EcAIII according to the cause of the lack of autoprocessing. Mutants marked in pink are not cleaved into subunits, while those marked in blue are mature but are unable to hydrolyze l-Asn.

However, due to the simultaneous multiple substitutions in the RDM1 series, it is not possible to discuss the contribution of each single mutation to the thermal stability of the entire EcAIII fold in detail, as the stabilizing/destabilizing effects of some substitutions might be complementary. On the other hand, it has previously been shown for *Flavobacterium meningosepticum* 2 aspartylglucosaminidase (fAGA), a structural homolog of EcAIII (Fig. 8 ▸ *a*), that even single substitutions in the active site, for example D183N, can significantly reduce the thermal stability (Liu *et al.*, 1998 ▸). These observations suggest that the EcAIII fold is flexible enough to accept different types of mutations while maintaining its structure and stability. This is a very promising feature for future EcAIII engineering.

### 4.2. Lesson 2: l-asparaginase activity of EcAIII is determined by the presence of Arg207

The l-asparaginase activity of EcAIII depends on the correct positioning of the substrate, which requires correlated interactions with several residues in the active site (Fig. 2 ▸). The experiments presented in this work show that Arg207 is absolutely critical for substrate binding in EcAIII. Its absence, with substitutions by Ala, Gly or other residues with small (Thr, Ser, Cys and Val), larger nonpolar (Ile and Pro) or polar (Gln and Asp) side chains, always led to a loss of l-asparaginase activity. Therefore, all variants analyzed in this work, without exception, were unable to hydrolyze l-asparagine, yielding negative Nessler results. However, it was demonstrated for fAGA that its variants, in which Arg180 (the counterpart of Arg207 in EcAIII) was replaced by Lys, Gln and Ile, can slowly be processed to the mature form (Fig. 8 ▸ *a*) and have a reduced, but not abolished, enzymatic activity (Liu *et al.*, 1998 ▸). These findings indicated that fAGA can bind its substrate in the absence of Arg180. However the substrate in this case is different from l-Asn. Moreover, as all EcAIII variants cleaved into subunits also carried other substitutions, the lack of l-asparaginase activity could be the cumulative effect of several mutations. Interestingly, this effect was always negative, as all analyzed combinations of residues were unable to stabilize the EcAIII substrate for its hydrolysis to l-Asp.

Activity tests of the new variants were always conducted together with a positive and a negative control (see Section 2). In the negative controls we never observed a yellow-orange color, despite the presence in the lysate of a physiological abundance of the natural *E. coli* l-asparaginases EcAI (class 1), EcAII (class 1) and EcAIII (class 2). This indicates that the physiological concentration of these enzymes is too low to interfere with the activity of the overproduced recombinant proteins. This observation opens the possibility for the design of more sophisticated experiments, for example directed evolution, that require high-throughput activity screening.

### 4.3. Lesson 3: autoprocessing does not require a strictly defined network of residues

As all of the variants described in this work have their l-asparaginase activity abolished by the absence of Arg207, their structures can be used to investigate the details of the maturation process. In general, the chemical character of the residues at positions 210 and 211 did not affect the autocatalytic maturation. Asp210 and Ser211 could be substituted individually (variant RDM1-37, D210S mutation only) or simultaneously (variants RDM1-3, RDM1-8, RDM1-12, RDM1-18, RDM1-24, RDM1-29 and RDM1-38) without any loss of autocatalytic activity. Moreover, the type of substitution (Table 1 ▸), *i.e.* polar to polar (RDM1-24), polar to non­polar (RDM1-12, RDM1-18 and RDM1-29) or polar to polar/nonpolar (mixed combination; RDM1-3, RDM1-8 and RDM1-38) also did not influence the ability to autocleave. Although the types of side chains present at positions 210 and 211 do affect the number of water molecules and the hydrogen-bond network in the close vicinity of the threonine triad, this pattern does not seem to affect the auto-maturation process, at least for the tested substitutions (Table 1 ▸).

Previous experiments revealed that substitutions in the active-site area of the EcAIII homolog HsAIII did affect the autoproteolytic activity (De Morais & De Souza, 2021 ▸). Substitution of the nucleophilic Thr by Ala completely abolished autocleavage (Li *et al.*, 2016 ▸), and the same effect was observed for EcAIII (Michalska *et al.*, 2008 ▸). Substitutions of the remaining threonines in the triad reduced the autocleavage rate of HsAIII, but processing could be restored with glycine (Nomme *et al.*, 2014 ▸; Li *et al.*, 2016 ▸). We also tested whether the addition of glycine might initiate autocleavage of the immature RDM1 variants, but the results were negative. When Arg196/207 (HsAIII/EcAIII) responsible for substrate anchoring was replaced by Gln in HsAIII (Li *et al.*, 2016 ▸), autoprocessing was still possible. Similarly, in our variant RDM1-8 the R207Q mutation did not abolish autocleavage.

Analysis of single substitutions at positions corresponding to Arg207 in other structural homologs of EcAIII (Fig. 8 ▸ *a*), in particular human and *F. meningosepticum* AGAs, revealed that this residue is important for autocleavage. In fAGA the single mutations R180K, R180Q and R180L (Fig. 8 ▸ *a*) significantly reduced the autoproteolysis rate (Xu *et al.*, 1999 ▸; Guan *et al.*, 1998 ▸). In human AGA (hAGA), the mutations R234Q, R234K and R234A (Fig. 8 ▸ *a*) resulted in misprocessing of the precursor (Tikkanen *et al.*, 1996 ▸). These data indicate that Arg207 plays a role in stabilization of the precursor structure. Our data suggests that some variants of EcAIII are able to mature even in the absence of Arg207 (Table 1 ▸), meaning that the negative effect of the absence of Arg207 is compensated by the presence of other simultaneous substitutions that stabilize the correct geometry of the precursor.

There are no data showing the role of Gly206 in the maturation process. As this residue is located next to Arg207 in a solvent-exposed loop and relatively far from the nucleophilic threonine in EcAIII (Fig. 1 ▸), it can be assumed that it plays a rather minor role in stabilizing the precursor. Only one study reported mutagenesis at a position corresponding to Ser211 in EcAIII. It was observed that the substitution S238A in hAGA (Fig. 8 ▸ *a*) delayed (but did not abolish) precursor processing (Saarela *et al.*, 2004 ▸).

Human AGA contains Asp237, which corresponds to Asp210 in EcAIII (Fig. 8 ▸ *a*). It has been demonstrated that a D237A mutant of hAGA had an impaired autocleavage rate, while the D237S variant was processed as the WT protein (Saarela *et al.*, 2004 ▸). Similarly, substitution of Asp184 by Ala (D183A) in fAGA (Fig. 8 ▸ *a*) reduced its autocleavage rate by half, while D183E and D183N mutations decreased the rate of autoprocessing only slightly (Liu *et al.*, 1998 ▸). Eventually, it was concluded that counterparts of Asp210 might play a role in correct protein folding and participate in maintaining the proper positions of the water molecules necessary for autocleavage (Saarela *et al.*, 2004 ▸; Liu *et al.*, 1998 ▸). These findings suggest that polar residues are preferred at position corresponding to Asp210 in EcAIII, while nonpolar residues, for example Ala, decrease the maturation rate (Fig. 8 ▸ *a*). However, such observations are not directly applicable to EcAIII, as different types of substitutions, among them the nonpolar Ala, Pro or Leu, were observed at position 210 in EcAIII variants that cleaved into subunits (Table 1 ▸).

### 4.4. Lesson 4: initiation of autoprocessing is a complex process

A literature survey allowed us to identify and classify several ‘checkpoints’ that Ntn-amidohydrolases must pass to undergo correct processing into subunits and develop enzymatic activity (Fig. 8 ▸ *b*). Efficient expression (1; the numbers refer to Fig. 8 ▸ *b*), correct folding (2) and proper dimerization (3) of the precursor are the first prerequisites for autocatalytic activation (Saarela *et al.*, 2004 ▸; Morais *et al.*, 2020 ▸; Li *et al.*, 2012 ▸). Proper folding together with efficient dimerization introduces torsional strain (4) at the scissile bond that is the driving force for initiation of the autocleavage reaction. Another factor is the positioning of the linker (or spacer peptide; 5) because it affects the conformation of the residues in the scissile-bond area (Hewitt *et al.*, 2000 ▸). A correct pose of the linker is also necessary for the positioning of water molecules (6) close to the nucleophilic Thr and the scissile bond (Michalska *et al.*, 2008 ▸; Saarela *et al.*, 2004 ▸; Liu *et al.*, 1998 ▸). In most Ntn-hydrolase structures there is one clearly selected water molecule (w1; Fig. 6 ▸ *a*) that is necessary for initiation of the autocatalytic cleavage. Removal of water w1 leads to structural rearrangements, resulting in rotation of the nucleophilic hydroxyl into a catalytically unfavorable position (Yoon *et al.*, 2004 ▸). Initiation of the autoprocessing attack also requires the correct positioning of the scissile bond (7; Nomme *et al.*, 2012 ▸, 2014 ▸; Michalska *et al.*, 2008 ▸; Li *et al.*, 2016 ▸) and of the side chain of the nucleophilic Thr (8). It has been shown that the nucleophilic Thr can fluctuate between ‘inactive’ (*trans*) and ‘active’ (*cis*) states, but this conversion requires proper flexibility of the active site and its close neighborhood, including appropriate arrangement of the residues forming the oxyanion hole (9; Buller *et al.*, 2012 ▸; Schmitzberger *et al.*, 2003 ▸). To initiate the nucleophilic attack, the side chain of the Thr nucleophile has to be polarized (10). It has been suggested that water w1 is responsible for this polarization (Yoon *et al.*, 2004 ▸); however, there are also reports showing that the nucleophilic threonine is activated by a spatially close Thr neighbor and not by a solvent molecule (Pica *et al.*, 2016 ▸).

To undergo autoproteolytic cleavage, an Ntn-hydrolase precursor must fulfill all of the requirements (pass the ‘checkpoints’ numbered 1–10 in Fig. 8 ▸ *b*). The observations collected in this list indicate that even small distortions of any element responsible for maintaining the fragile architecture of the autoproteolytic center can abolish or significantly impair the autocleavage process. As shown in Fig. 8 ▸(*b*), the mutations introduced in the autoprocessed RDM1 variants (Table 1 ▸) were not harmful to the sensitive apparatus, and even the large side chain of Trp211 in variant RDM1-18 (Fig. 5 ▸), located in the close proximity of the nucleophilic Thr179, did not perturb the autoprocessing. However, these variants were enzymatically inactive (Fig. 8 ▸ *b*), despite the fact that in all of the mature variants (except RMD1-18; Fig. 6 ▸ *c*) there is still room for substrate binding. The absence of Arg207 together with the mutated positions 210 and 211 are most likely to be responsible for poor substrate stabilization in the active site.

Analysis of the pre-maturation requirements (Fig. 8 ▸ *b*) offers a possible explanation as to why some of the RDM1 mutants were not processed into subunits. As indicated by SEC (Supplementary Fig. S4) and CD spectra (Supplementary Fig. S3), mutant RDM1-42 was produced in a soluble form but carried structural distortion (due to impaired folding) and had a tendency to aggregate (impaired dimerization). Modeling of its precursor structure suggested that this distortion might be a consequence of the G206P mutation, which affects the conformation of the entire 200–208 loop (Supplementary Fig. S5). The predicted structures of precursors RDM1-41, RDM1-61 and RDM1-63 (Fig. 7 ▸) indicated that autoprocessing of these variants was abolished by incorrect positioning and polarization of Thr179 (Fig. 8 ▸ *a*). While the modeling did not suggest any important changes in the active site of the RDM1-43, RDM1-46 and RDM1-64 mutants (Supplementary Fig. S5), it can be assumed that they were correctly folded and had proper quaternary structure (Supplementary Figs. S3 and S4); thus, the reasons for aborted autoprocessing are probably related to incorrect positioning of the linker and scissile bond or an incorrect arrangement of water molecules or of the oxy­anion hole. As the exact role of the intrinsically disordered linker is not clear at present (Loch & Jaskolski, 2021 ▸), its impact on autoprocessing should be investigated in detail in future experiments. To date, there are no crystallographic data that allow a comprehensive analysis of the conformation of the linker: even in the structures of the uncleaved EcAIII mutant T179A (PDB entry 3zal) or of uncleaved HsAIII (PDB entry 4osx) a large portion of the linker could not be traced in the electron-density maps (Michalska *et al.*, 2008 ▸; Su *et al.*, 2013 ▸).

All of these findings indicate that initiation of the autocatalytic maturation is a very complex process. Our results demonstrate that even mutations that seem to appear to be unrelated to autocleavage can dramatically alter the enzyme performance in this process. These observations are very important for future engineering of EcAIII, as mutations within the binding pocket that might potentially improve the l-asparaginase activity could also permanently abolish the autocatalytic activation of the enzyme. The results discussed above agree with the observations reported earlier that the autoproteolytic and l-asparaginase activities are separate molecular events (Li *et al.*, 2016 ▸). While the l-asparaginase activity is developed only as a consequence of autocleavage (Fig. 8 ▸ *b*), the autoprocessing is independent of the l-asparaginase activity and can also occur efficiently in variants that have dysfunctional apparatus for substrate binding and/or catalysis.

### 4.5. Lesson 5: EcAIII can adopt conformational states similar to potassium-dependent enzymes

In most of the analyzed structures Arg207 was replaced by a smaller residue, and this was accompanied by a conformational change of Glu234 and a shift of His119 (Fig. 4 ▸). These movements resemble the potassium-induced changes observed in the potassium-dependent class 2 l-asparaginase from *Phaseolus vulgaris*, PvAIII(K) (Bejger *et al.*, 2014 ▸). In the structure of PvAIII(K), in addition to the stabilization loop (or sodium-binding loop), there is another alkali-metal-binding loop (activation loop) in subunit α (Val111–Ser118). The activation loop acts as a ‘catalytic switch’ triggered by alkali-metal exchange. Coordination of the K^+^ cation switches the enzyme to the (catalytically active) ON state. However, when the smaller Na^+^ cation is coordinated the enzyme is reset to the (catalytically inactive) OFF state (Bejger *et al.*, 2014 ▸).

**Figure 9:**
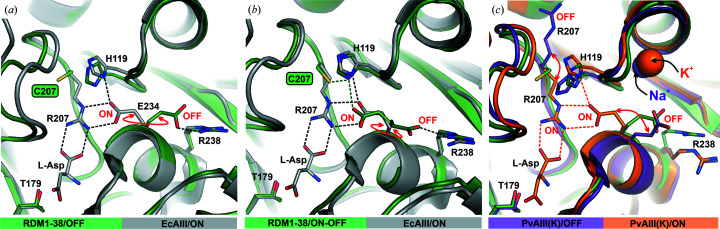
Structures of the enzyme in the ON/OFF states. (*a*) Superposition of WT EcAIII in the ON state, ready to coordinate a substrate molecule (gray, PDB entry 2zal), and mutant RDM1-38 (green) in the OFF state, indicated by rotation of Glu234. (*b*) The second subunit β of the RDM1-38 structure with Glu234 in two alternative orientations typical of the ON/OFF states; Glu234 in the ON state is locked by hydrogen bonds to His119 and Cys207. (*c*) Superposition of mutant RDM1-38 (green) in the OFF state and the structures of PvAIII(K) in the ON (orange, PDB entry 4pu6) and OFF (violet, PDB entry 4pv3) states. In all panels, the mutation sites are marked in frames and hydrogen bonds are marked as dashed lines.

Superposition of the PvAIII(K) structure in the ON (PDB enty 4pu6) and OFF (PDB entry 4pv3) states with the structures of the RDM1 variants revealed that the conformational rearrangements in the EcAIII mutants resemble the movement of residues in the potassium-dependent enzyme, leading to switching between the ON and OFF states. In WT EcAIII Glu234 is hydrogen-bonded to Arg207, locking it together with His119 in the position required to coordinate the substrate (as in the ON state; Fig. 9 ▸). The same orientation of Glu234 was observed in one subunit of the RDM1-8 variant (Fig. 4 ▸ *f*), while in all other variants the alternative orientation of Glu234 was found (Fig. 4 ▸). This orientation is the same as the OFF orientation of the PvAIII(K) counterpart Glu250. In the OFF state Glu250 moves away from the active site, while His117, like its EcAIII counterpart His119, moves in to block the active site.

These observations indicate that EcAIII can also be set to the OFF state; however, this state is enforced by genetic amputation of Arg207, which is crucial for substate binding. Although the counterpart of the activation loop (residues 113–120) also exists in EcAIII, it is not able to coordinate metal ions (Ajewole *et al.*, 2018 ▸). As Arg238 seems to be crucial for stabilizing the OFF state in the catalytically inactive RDM1 variants, the question arises as to whether EcAIII can also be switched between the ON and OFF states. The trigger could not be alkali-metal coordination but could be another factor, for example pH, that changes the ionization state of Glu234 and His119. As EcAIII is active at pH of about 8, this might be a mechanism that affects its pH-dependent activity. This speculation, however, would require further experimental validation.

### 4.6. Lesson 6: the EcAIII subunits are not acting equivalently

Detailed analysis of the RDM1 structures showed that the structural changes are not equivalent in the two parts of the dimer. The differences varied between the mutants but are most visible in the following three regions: the α-helix residues 147–159 in front of the flexible linker, the loop 200–208 carrying residues necessary for substrate binding and the loop 116–120 with His119. The differences are visible, for example, in the structure of variant RDM1-8 (Supplementary Fig. S7), where the pattern of hydrogen bonds involving Gln207 differs in the two subunits (Figs. 4 ▸ *e* and 4 ▸ *f*). In this case, the pair of residues that are responsible for the structural differences are His119 and Glu234. As indicated in Figs. 2 ▸ and 9 ▸, His119, which together with Glu234 is responsible for the stabilization of Arg207, is contributed by another subunit of the dimer. If Glu234 adopts the OFF conformation in one subunit, this is associated with movement of His119 and the entire 116–120 fragment in the second subunit.

In other variants, for example RDM1-12, differences are visible in the regions of α-helix 147–159, loop 200–208 and loop 116–120, while in RDM1-38 differences are only observed in the region of α-helix 147–159 (Supplementary Fig. S7). Although slightly different subunit conformations might also arise from crystal packing (which is unlikely in the isomorphous RDM1 series), it seems that these differences are not accidental: in all crystal structures of EcAIII deposited in the PDB there is always at least one complete dimer in the asymmetric unit. This may indicate that the subunits are intrinsically non-equivalent and thus evade crystallographic symmetry.

## 5. Conclusions

We used local random mutagenesis, X-ray crystallography, CD spectroscopy, nanoDSF and molecular modeling to study the catalytic apparatus of EcAIII, a model Ntn-hydrolase and class 2 l-asparaginase. The genetic modifications of the EcAIII sequence revealed that the enzyme mechanism is very sensitive to substitutions and that successful autoproteolytic activation depends on a number of factors. Some of these factors were already known (Fig. 8 ▸ *b*), but many have not yet been characterized. Class 2 l-asparaginases can potentially be engineered for clinical applications as antileukemic agents, and this may open new possibilities for the design of innovative treatment protocols. In our experiments highly thermostable EcAIII variants were created, showing that the EcAIII fold is robust and accommodates various residue substitutions well. The lessons presented in this work could be considered when designing further variants of EcAIII with ALL therapy in sight.

## 6. Databases

Atomic coordinates and structure factors corresponding to the final models have been deposited in the Protein Data Bank (PDB) under the accession codes 7qy6 (WT EcAIII), 7qtc (RDM1-3), 7qq8 (RDM1-8), 7qsf (RDM1-12), 7qym (RDM1-18), 7qyx (RDM1-24), 7r5c (RDM1-29), 7qvr (RDM1-37) and 7r1g (RDM1-38). The corresponding raw X-ray diffraction images have been deposited in the MX-RDR Repository at the Interdisciplinary Centre for Mathematical and Computational Modeling (ICM) of the University of Warsaw with the following digital object identifiers (DOIs): https://doi.org/10.18150/VAOJLJ (WT EcAIII), https://doi.org/10.18150/R8VJ7V (RDM1-3), https://doi.org/10.18150/VAZZ2F (RDM1-8), https://doi.org/10.18150/T0WC49 (RDM1-12), https://doi.org/10.18150/LIENZ5 (RDM1-18), https://doi.org/10.18150/6OXPLO (RDM1-24), https://doi.org/10.18150/WEFSC9 (RDM1-29), https://doi.org/10.18150/R3BTBM (RDM1-37) and https://doi.org/10.18150/XSEXUF (RDM1-38).

## Supplementary Material

PDB reference: EcAIII, wild type, 7qy6

PDB reference: RDM1-3, 7qtc

PDB reference: RDM1-8, 7qq8

PDB reference: RDM1-12, 7qsf

PDB reference: RDM1-18, 7qym

PDB reference: RDM1-24, 7qyx

PDB reference: RDM1-29, 7r5c

PDB reference: RDM1-37, 7qvr

PDB reference: RDM1-38, 7r1g

Supplementary Figures. DOI: 10.1107/S2059798322005691/jv5010sup1.pdf

Diffraction data for WT EcAIII: https://doi.org/10.18150/VAOJLJ

Diffraction data for RDM1-3: https://doi.org/10.18150/R8VJ7V

Diffraction data for RDM1-8: https://doi.org/10.18150/VAZZ2F

Diffraction data for RDM1-12: https://doi.org/10.18150/T0WC49

Diffraction data for RDM1-18: https://doi.org/10.18150/LIENZ5

Diffraction data for RDM1-24: https://doi.org/10.18150/6OXPLO

Diffraction data for RDM1-29: https://doi.org/10.18150/WEFSC9

Diffraction data for RDM1-37: https://doi.org/10.18150/R3BTBM

Diffraction data for RDM1-38: https://doi.org/10.18150/XSEXUF

## Figures and Tables

**Table 1:** RDM1 variants generated by random mutagenesis The variants are classified according to their ability to autoprocess. Thermal stability (*T*_m_) was assessed by nanoDSF. Mutants with significantly increased thermal stability are highlighted by bold *T*_m_ values.

	Mutation site				
Variant	206	207	210	211	*T*_m_ (°C)
Ability to process into α/β subunits					
WT	**G**	**R**	**D**	**S**	69.0
RDM1-3	H	T	P	Q	64.5
RDM1-8	Y	Q	P	T	59.7
RDM1-12	C	T	A	A	**72.9**
RDM1-18	—	V	P	W	**75.3**
RDM1-24	—	A	S	T	**83.3**
RDM1-29	C	S	L	V	65.9
RDM1-37	S	T	S	—	64.3
RDM1-38	—	C	S	V	**74.5**
No ability to process into α/β subunits					
RDM1-41	P	T	M	R	54.4
RDM1-42	P	I	T	P	53.9
RDM1-43	V	G	S	—	55.8
RDM1-46	L	D	L	—	57.7
RDM1-61	A	P	S	E	53.6
RDM1-63	S	G	G	R	57.2
RDM1-64	I	V	I	G	57.6

**Table 2:** Statistics of data collection and structure refinement (variants capable of processing into α/β subunits) Values in parentheses are for the highest resolution shell.

Structure	WT	RDM1-3	RDM1-8	RDM1-12	RDM1-18	RDM1-24	RDM1-29	RDM1-37	RDM1-38
Data collection									
Radiation source	P13, EMBL/DESY	Synergy-S	Synergy-S	Synergy-S	P13, EMBL/DESY	P13, EMBL/DESY	Synergy-R	Synergy-S	Synergy-S
Wavelength (Å)	0.97000	1.54056	1.54056	1.54056	0.77490	0.77490	1.54178	1.54056	1.54178
Space group	*P*2_1_2_1_2_1_	*P*2_1_2_1_2_1_	*P*2_1_2_1_2_1_	*P*2_1_2_1_2_1_	*P*2_1_2_1_2_1_	*P*2_1_2_1_2_1_	*P*2_1_2_1_2_1_	*P*2_1_2_1_2_1_	*P*2_1_2_1_2_1_
*a*, *b*, *c* (Å)	50.48, 75.35, 147.99	51.35, 75.04, 149.03	50.00, 74.86, 147.84	51.62, 75.05, 149.14	49.55, 75.01, 146.95	50.25, 74.14, 147.47	51.87, 78.00, 148.72	51.22, 74.11, 149.77	49.82, 74.69, 147.36
Resolution range (Å)	75.35–1.55 (1.64–1.55)	20.36–2.55 (2.66–2.55)	19.16–1.80 (1.84–1.80)	19.05–1.60 (1.63–1.60)	66.90–1.20 (1.22–1.20)	52.34–1.85 (1.95–1.85)	21.48–2.20 (2.27–2.20)	20.28–1.90 (1.94–1.90)	21.13–1.95 (2.00–1.95)
Reflections collected	1018784	49515	126617	157631	2303211	632143	136626	111466	104990
Unique reflections	83105 (13084)	17974 (2215)	48975 (2945)	69731 (3274)	167749 (8018)	48066 (7553)	30380 (2652)	42970 (2773)	38942 (2596)
Completeness (%)	99.5 (98.0)	92.7 (95.3)	93.6 (95.5)	90.7 (86.8)	98.1 (95.7)	98.4 (97.7)	97.6 (99.2)	95.6 (89.8)	95.3 (90.7)
Multiplicity	12.2 (12.1)	2.8 (2.5)	2.6 (2.1)	2.3 (1.8)	13.7 (13.2)	13.1 (12.4)	4.5 (4.8)	2.6 (2.1)	2.7 (2.2)
*R*_merge_ (%)	16.7 (139.0)	11.9 (67.6)	7.6 (51.9)	5.6 (22.0)	8.5 (178.6)	11.9 (214.1)	12.4 (49.6)	7.9 (37.2)	9.9 (36.1)
*R*_meas_ (%)	15.8 (123.7)	14.3 (83.0)	9.2 (63.1)	7.0 (29.3)	8.8 (185.7)	11.8 (185.2)	14.0 (55.5)	9.7 (48.7)	12.1 (46.4)
〈*I*/σ(*I*)〉	9.36 (1.62)	7.20 (2.10)	8.00 (1.60)	9.80 (3.00)	14.20 (1.70)	15.16 (1.75)	6.4 (1.90)	9.00 (2.10)	7.00 (2.10)
CC_1/2_ (%)	99.7 (90.4)	98.3 (60.4)	99.6 (69.2)	99.5 (88.3)	99.9 (55.6)	99.9 (78.9)	99.2 (90.2)	99.2 (70.2)	99.1 (74.1)
Structure refinement									
No. of reflections									
Unique	81414	16922	47923	68661	166598	46910	29315	41874	37806
Test set	1079	1010	1001	1039	1086	1031	1039	1055	1020
*R*_work_/*R*_free_ (%)	19.3/20.5	20.2/25.1	19.5/23.6	17.4/21.1	12.7/15.3	19.8/23.9	24.9/30.1	22.3/28.8	24.8/28.3
No. of atoms									
Protein	4235	4025	4254	4237	4450	4079	4203	4286	4204
Solvent	4031	142	414	598	617	208	107	543	171
ADP (Å^2^)									
Protein	29.90	36.31	18.97	12.60	19.35	35.23	40.13	15.30	16.23
Solvent	27.10	17.47	28.02	22.70	37.63	43.02	35.57	22.22	19.35
R.m.s.d.									
Bond lengths (Å)	0.010	0.009	0.011	0.010	0.013	0.011	0.011	0.011	0.009
Angles (°)	1.497	1.587	1.609	1.672	1.727	1.568	1.622	1.657	1.617
Ramachandran plot (%)									
Favored	98	97	97	97	98	97	98	97	97
Allowed	2	3	3	3	2	3	2	3	3
Outliers	0	0	0	0	0	0	0	0	0
PDB code	7qy6	7qtc	7qq8	7qsf	7qym	7qyx	7r5c	7qvr	7r1g
Diffraction data DOI	https://doi.org/10.18150/VAOJLJ	https://doi.org/10.18150/R8VJ7V	https://doi.org/10.18150/VAZZ2F	https://doi.org/10.18150/T0WC49	https://doi.org/10.18150/LIENZ5	https://doi.org/10.18150/6OXPLO	https://doi.org/10.18150/WEFSC9	https://doi.org/10.18150/R3BTBM	https://doi.org/10.18150/XSEXUF

## Footnotes

1 Current taxonomic classification: *Dickeya dadantii*.

2 Alternative names: *Elizabethkingia meningoseptica* and *E. miricola*.
