# Supplementary material for: Structural and biophysical studies of new l-asparaginase variants: lessons from random mutagenesis of the prototypic Escherichia coli Ntn-amidohydrolase
Source: Acta Crystallogr D Struct Biol. 2022 Jun 28;78(Pt 7):911–26. doi: 10.1107/S2059798322005691 (PMC9248843; doi:10.1107/S2059798322005691)
Supplement: Supplementary file 1 [file d-78-00911-sup1.pdf]

STRUCTURAL
BIOLOGY

Volume 78 (2022)

Supporting information for article:

Structural and biophysical studies of new l-asparaginase variants: lessons from random mutagenesis of the prototypic *Escherichia coli* Ntn-amidohydrolase

Joanna I. Loch, Agnieszka Klonecka, Kinga Kądziołka, Piotr Bonarek, Jakub Barciszewski, Barbara Imiolczyk, Krzysztof Brzezinski, Mirosław Gilski and Mariusz Jaskolski

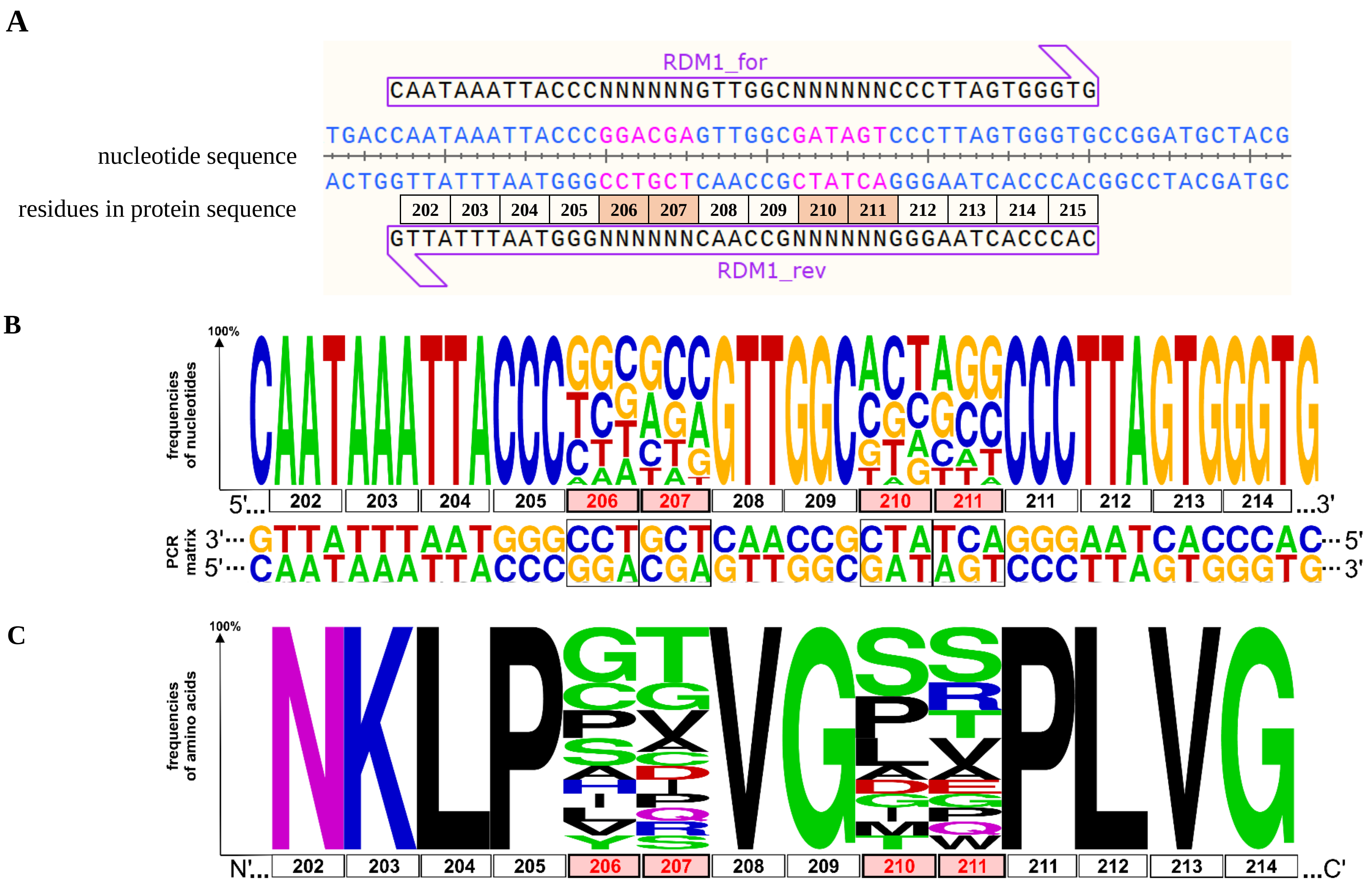

Fig. S1. (A) Detailed sequence of the designed degenerate primers (N - random nucleotide) and their annealing to the PCR matrix. Nucleotides marked in pink encode amino acid residues at positions: 206, 207, 210 and 211, marked in orange boxes. (B) Graphical representation of the frequency of nucleotide substitutions in codon positions 206, 207, 210 and 211, (C) and the corresponding frequency of protein amino acid types. Nucleotides are colored according to their types: A (green), T (red), C (blue) and G (yellow). Amino acids are colored according to their chemical character: KRH (blue), DE (red), NQ (magenta), SCT (orange), YW (pink), G (black) and AVLIPM (green).

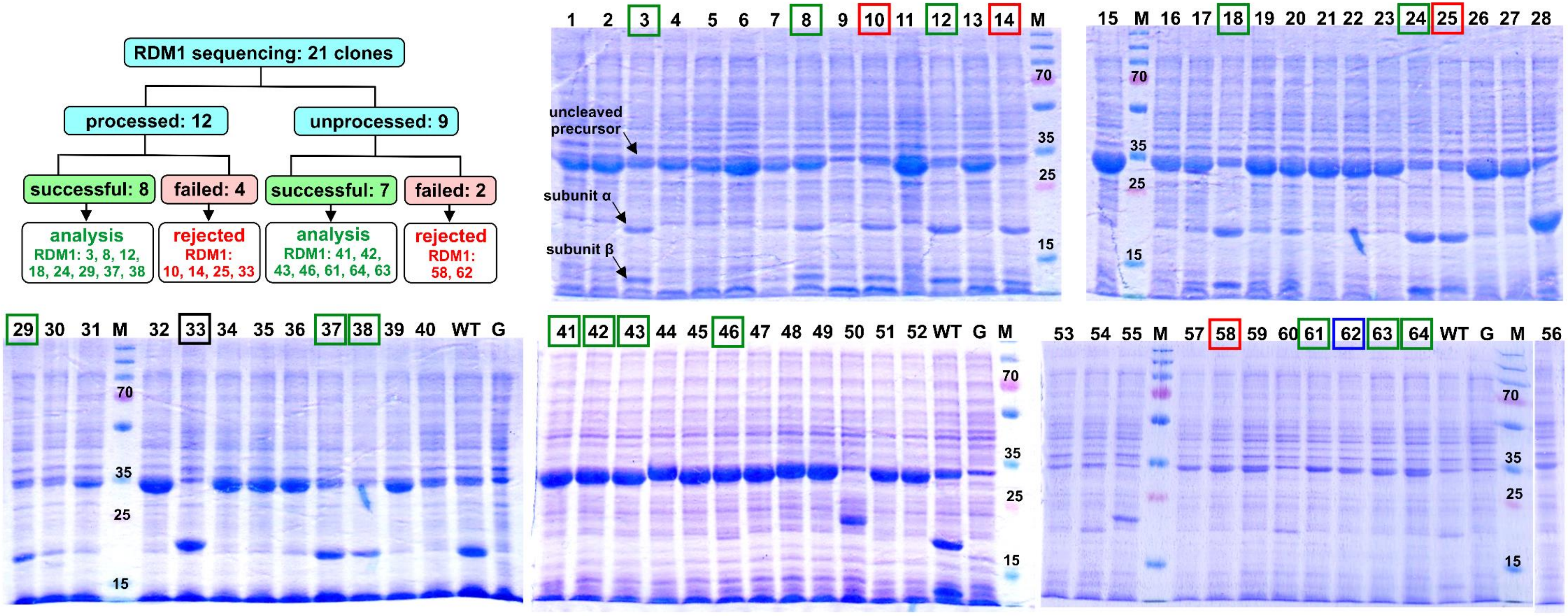

Fig. S2. SDS-PAGE gels after small-scale expression: selection of the clones after locally performed random mutagenesis (RDM1). Most of the clones were not processed for subunits α/β. The chart in the left top corner shows the events leading to the selection of proteins characterized in this work: 21 clones were selected for sequencing. Sequencing was successful for 15 variants (8 cleaved and 7 unprocessed, green boxes with gel lane numbers). Sequencing failed for clones 10, 14, 25 and 58 (no annealing of the T7 sequencing primers to the matrix; red boxes with gel lane numbers). One clone (33, marked by black box) had an abortive STOP codon at position 207. In the case of clone 62 (blue box), a mixture of plasmids was identified with substitutions: 206 L/M, 207 A/S, 210 L/N, 211 R/R.

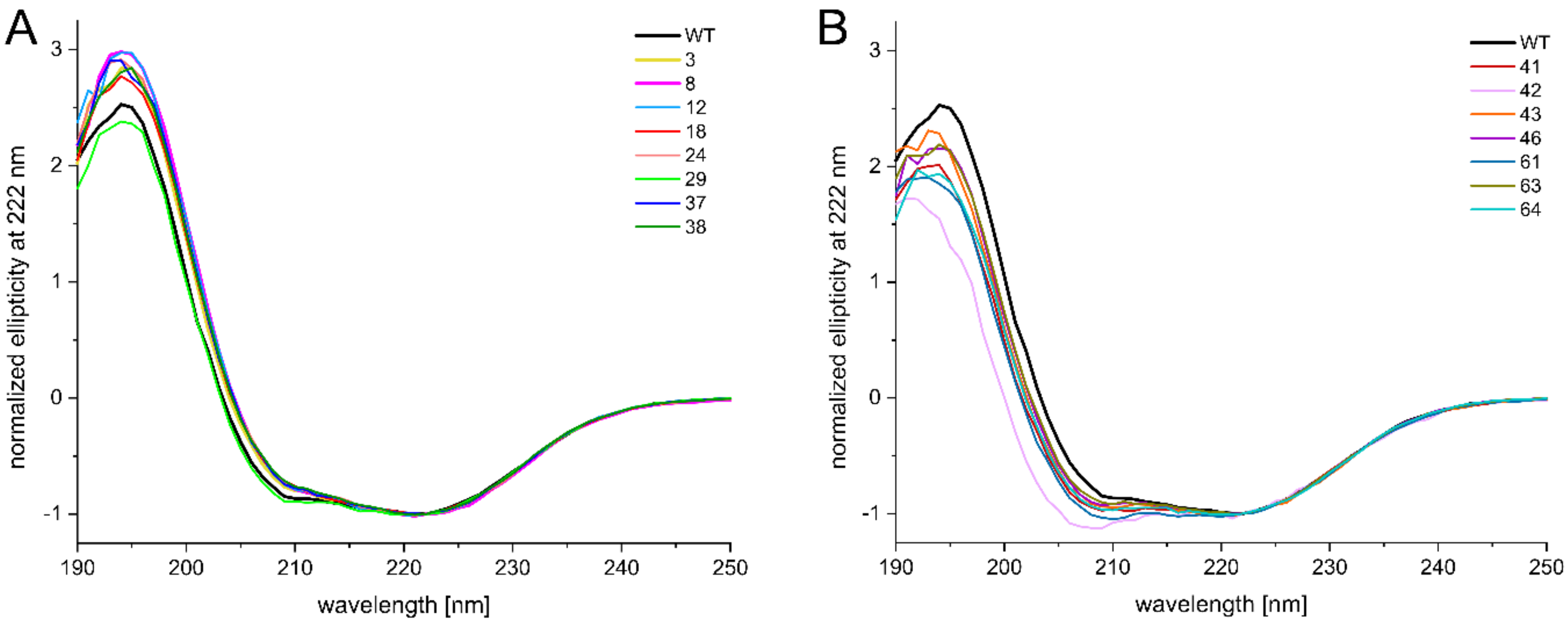

Fig. S3. Far-UV CD spectra recorded for EcAIII variants. The variants are marked by color according to their number in Table 1. (A) Mutants processed for subunits α/β; (B) mutants incapable of autoprocessing.

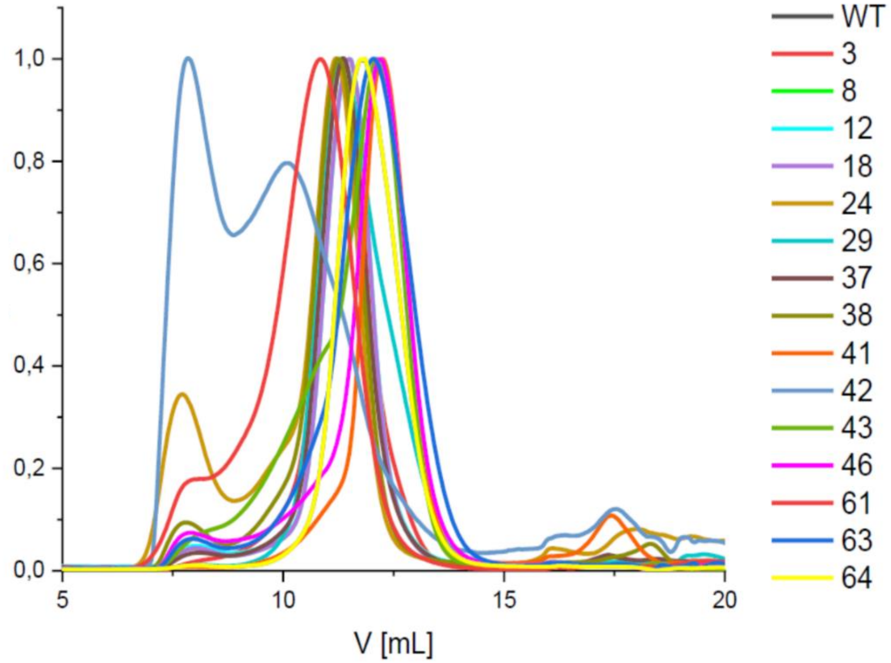

Fig. S4. Normalized chromatograms from size exclusion chromatography (SEC) using a Superdex75 10/300 GL column (GE Healthcare) obtained for RDM1 variants.

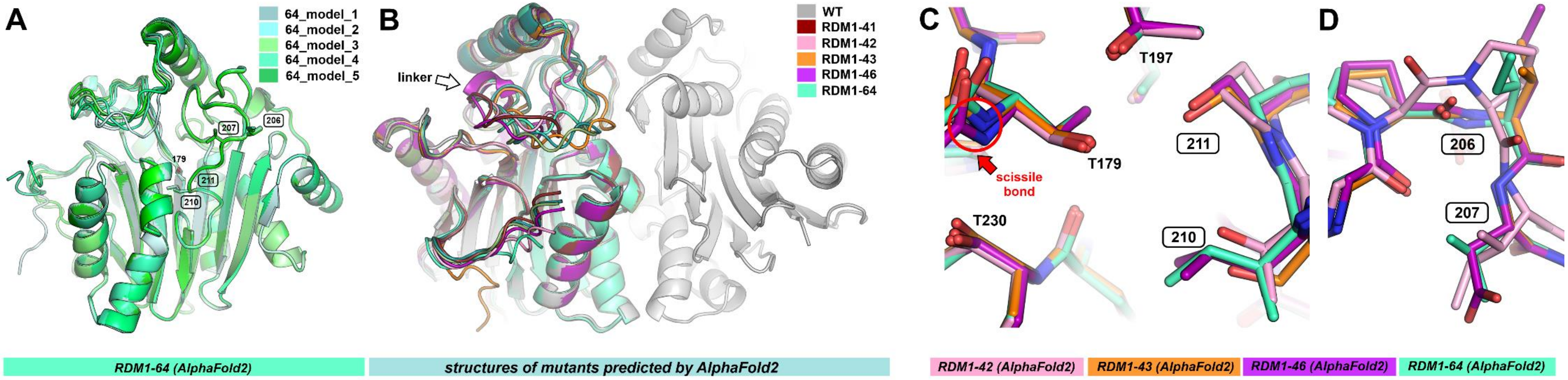

Fig. S5. (A) Superposition of the five models obtained for mutant RDM1-64. (B) Superposition of AlphaFold2 predicted "monomeric" structures of all RDM1 variants unprocessed for subunits. (C) Conformation of residues 210 and 211 in variants RDM1-42, 43, 46 and 64. (D) Position and conformation of residues 206 and 207 in RDM1-42, 43, 46 and 64. The color legend is presented in panel A and below panels B and C.

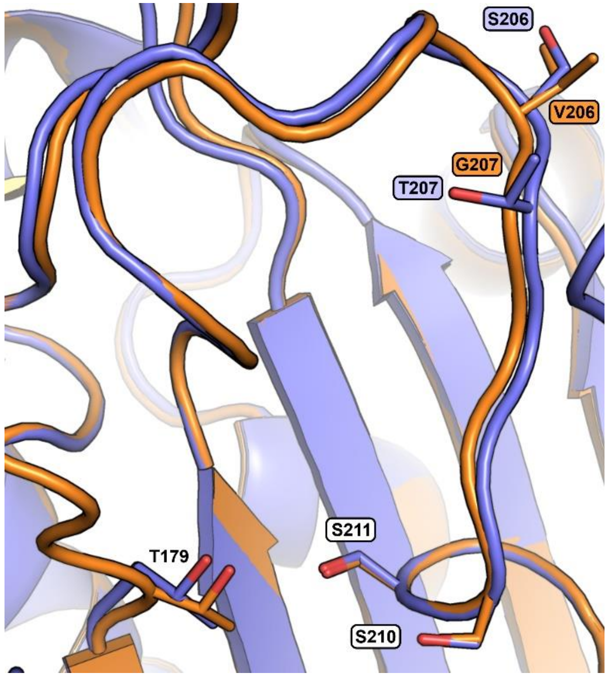

Fig. S6. Superposition of the AlphaFold2 predicted model of the uncleaved variant RDM1-43 (orange) and the crystal structure of variant RDM1-37, cleaved for subunits. Both variants have the same substitutions at positions 210 and 211. The fragment harboring residues 210-211 has almost identical conformation in the two structures. Some minor differences are visible in the region of substitutions 206-207.

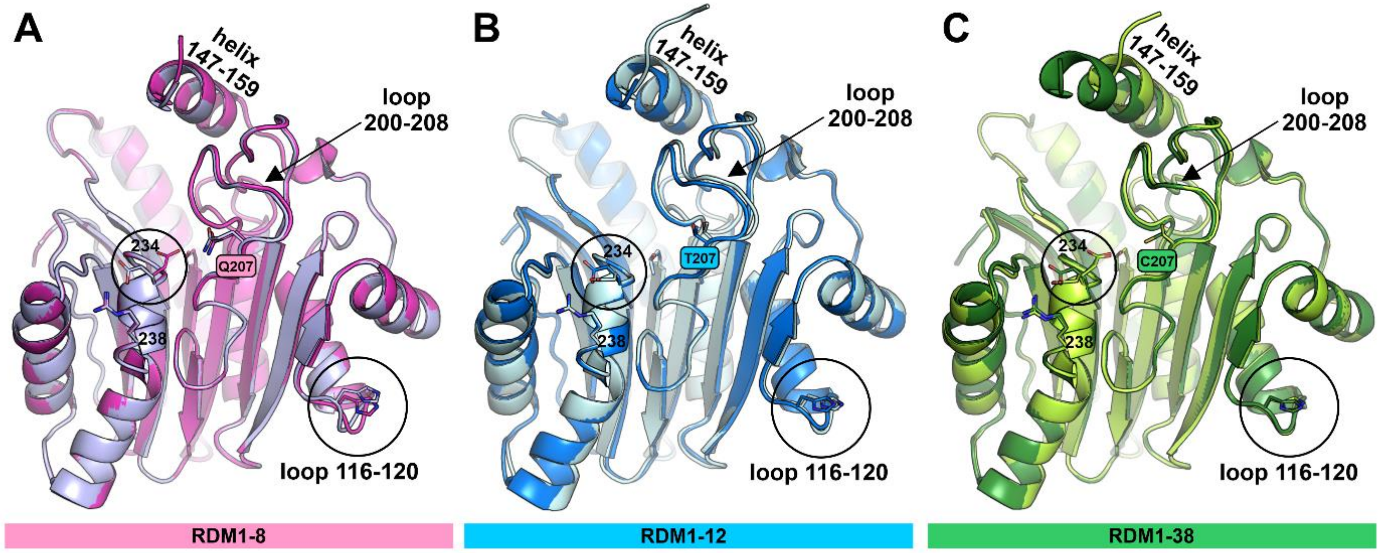

Fig. S7. Superposition of the dimer subunits of EcAIII mutants. Subunits (α+β) of RDM1-8 (A), RDM1-12 (B) and RDM1-38 (C); the second structural (α+β) unit of the dimer is shown in light color. In the structure RDM1-8 (A), the most significant differences are visible in the conformation of loop 116-120. In RDM1-12 (B), differences in the position of α-helix 147-159 and loops 116-120 and 200-208 can be detected. In RDM1-38 (C), loops 116-120 and 200-208 have similar conformation in both subunits, while the position of α-helix 147-159 differs significantly. In all panels, the mutation site 207 is marked in frame.
